# Assessing the Utility of a Cosmetic Dermatology Journal Club

**DOI:** 10.1111/jocd.70798

**Published:** 2026-04-13

**Authors:** Joycie Chang, Saira Alvi, Elizabeth Zhang, Sabrina S. Smith, Thanvi Gullapalli, Vanessa J. Lazaro‐Camp, Doroteja Dragovic, Areeba Ahmed, Drake C. Carter, Dawson E. Mills, Asia A. Henderson, Hanin El‐Khateeb, Bianca Y. Kang, Murad Alam

**Affiliations:** ^1^ Department of Dermatology Northwestern University Feinberg School of Medicine Chicago Illinois USA; ^2^ Department of Dermatology Mayo Clinic Scottsdale Arizona USA; ^3^ Department of Surgery Northwestern University Feinberg School of Medicine Chicago Illinois USA; ^4^ Department of Otolaryngology Northwestern University Feinberg School of Medicine Chicago Illinois USA; ^5^ Department of Medical Social Sciences Northwestern University Feinberg School of Medicine Chicago Illinois USA

**Keywords:** clinical research, cosmetic dermatology, journal club, survey

## Abstract

**Background:**

Journal clubs provide opportunities for discussion of new and evolving practices and technologies.

**Objective:**

We sought to evaluate the utility and effectiveness of a monthly web‐based cosmetic dermatology journal club facilitated by faculty, residents, and medical students.

**Results:**

From May 2025 to September 2025, 16 articles were discussed and 41 responses were recorded (response rate: 60%). Most respondents found the journal club was an appropriate length, engaging, and very useful. Relevance, frequency, and likelihood to participate in the future varied significantly by participant type. Medical students and residents collectively considered the journal club too infrequent (*p* = 0.03) and reported it had less relevance to their practice (*p* < 0.001).

**Conclusion:**

A monthly web‐based cosmetic dermatology journal club was generally positively received by respondents.

## 1 Introduction

Cosmetic dermatology is a rapidly evolving field where practitioners need to regularly appraise new literature to ensure they are treating patients effectively. We are unaware of prior evaluations assessing the utility of cosmetic dermatology journal clubs.

The Association of Academic Cosmetic Dermatology (AACD) Journal Club holds monthly, web‐based journal clubs facilitated by faculty, fellows, residents, and medical students. For each session, faculty presenters select 4–5 articles regarding cosmetic and laser dermatology, which are presented by medical students and residents. Each presenter summarizes their selected article, and this is followed by a discussion. Most journal clubs are facilitated by a faculty‐to‐student ratio of 1:2. Faculty members consist of any member of the AACD, who are primarily clinical faculty. Although encouraged to attend, the journal club is not a required activity for residents or medical students.

## 2 Methods

Anonymous surveys regarding participant satisfaction were distributed via email to all attendees of the journal club, who included AACD faculty as well as residents and medical students (Table 1). Participant opinions were measured using a five‐point Likert scale, with values near five denoting a positive opinion. For questions about frequency, length, and number of articles, a score of three denoted a positive opinion. Responses were collected from May 2025 until September 2025.

**TABLE 1:** Survey sent to attendees of AACD journal clubs.

Question	Response options
What is your current role?	Medical student
	Dermatology resident
	Dermatology fellow
	Dermatology faculty
	Other (please specify)
Approximately how many AACD journal club sessions have you attended?	1
	2–3
	4–5
	More than 5
In your opinion, the length of the program was sufficient	1 = very short
	2 = short
	3 = neither too short nor too long
	4 = too long
	5 = much too long
In your opinion, the frequency of the program was sufficient	1 = much too infrequent
	2 = slightly too infrequent
	3 = neither too frequent nor infrequent
	4 = slightly too frequent
	5 = much too frequent
In your opinion, the number of articles discussed at each session was sufficient	1 = far too few
	2 = slightly too few
	3 = the right amount
	4 = slightly too many
	5 = far too many
In your opinion, the journal club is sufficiently interactive and engaging for all participants	1 = strongly disagree
	2 = disagree
	3 = neutral
	4 = agree
	5 = strongly agree
In your opinion, journal club presenters were appropriate and well qualified	1 = strongly disagree
	2 = disagree
	3 = neutral
	4 = agree
	5 = strongly agree
How relevant to your practice do you find the articles presented in the AACD journal club?	1 = very irrelevant
	2 = irrelevant
	3 = neither relevant nor irrelevant
	4 = relevant
	5 = very relevant
Overall, how useful do you find the AACD journal clubs?	1 = not at all useful
	2 = somewhat useful
	3 = neither useful nor not useful
	4 = useful
	5 = very useful
How likely are you to participate in this journal club activity in the future?	1 = very unlikely
	2 = unlikely
	3 = neutral
	4 = likely
	5 = very likely
How likely are you to recommend the AACD journal club to others?	1 = very unlikely
	2 = unlikely
	3 = neutral
	4 = likely
	5 = very likely
How interested would you be in presenting in a future journal club?	1 = very uninterested
	2 = uninterested
	3 = neither interested nor uninterested
	4 = slightly interested
	5 = very interested
Have any of the articles presented altered your practice of medicine? If so, which articles or on what subject?^a^	
Have any of the articles presented altered your training of residents/fellows? If so, which articles?^a^	

^a^ Free text response.

### 2.1 Data Analysis

Survey responses were recorded and compiled. Survey responses were averaged (means), and descriptive statistics were calculated. Statistical analyses were performed using R version 4.5.1 [1]. Comparisons of responses by participant type were performed using the non‐parametric Wilcoxon rank‐sum test. Participants of type “other” were excluded from subgroup analyses. A *p*‐value of < 0.05 was considered statistically significant.

## 3 Results

**FIGURE 1:**
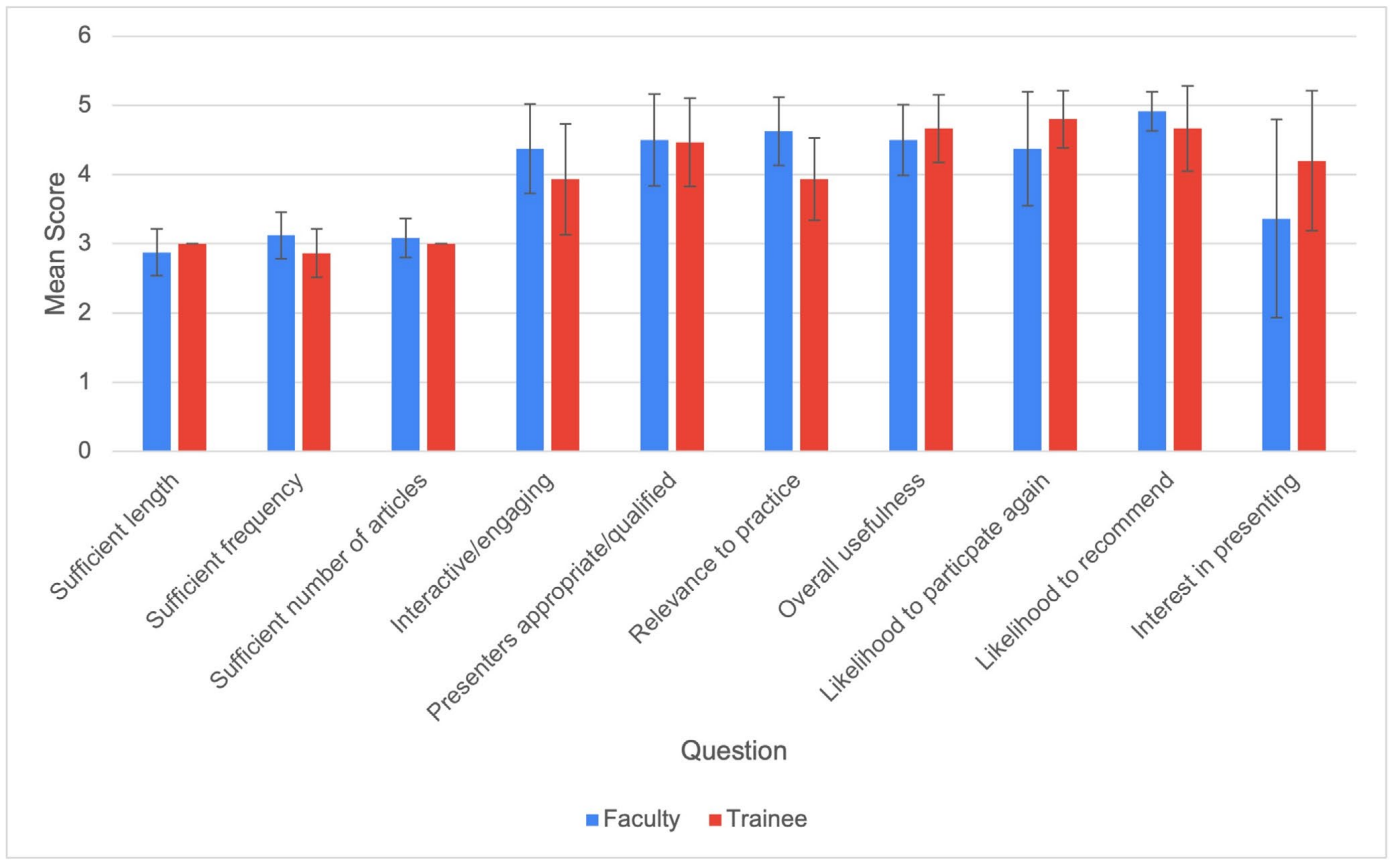
Responses by participant type. Mean score by faculty versus trainee (medical students and residents) participants. Blue bars indicate responses from faculty, and red bars indicate responses from trainees. Error bars indicate ±1 SD.

Sixteen articles were discussed during the specified time period, and 41/68 (60%) survey responses were recorded. Most respondents were dermatology faculty (59%), with medical students (20%), dermatology residents (17%), and others (5%) constituting the remainder. Over half (59%) of respondents attended at least four journal club sessions. The mean usefulness score was 4.6, and most respondents thought the length and frequency of the program were neither too long nor too short (mean score = 2.9) and neither too frequent nor too infrequent (mean score = 3.0), respectively. Most respondents felt that the number of articles discussed was the right amount (mean score = 3.0) and that articles were relevant to their practice (mean score = 4.4) (Table 2). Article topics that respondents reported altering their clinical practice included melasma management, lasers in skin of color, regenerative medicine, and combination injectables and lasers. Ninety‐two percent were somewhat or extremely likely to participate in another journal club, and 98% were somewhat or extremely likely to recommend the journal club to others. When analyzed by participant type, medical students and residents collectively considered the journal club too infrequent (*p* = 0.03) and felt it had less relevance to their practice (*p* < 0.001) (Table 2, Figure 1). Effect sizes (*r*) were 0.34 and 0.54, indicating medium and large effects, respectively.

**TABLE 2:** Responses by participant type.

Question	Mean score ± Standard deviation			*p* ^c^
	Overall (*N* = 41)	Medical students and residents (*N* = 24)	Faculty (*N* = 15)	
Length of program sufficient^a^	2.92 ± 0.26	3.00 ± 0.00	2.88 ± 0.34	0.16
Frequency of program sufficient^a^	3.02 ± 0.35	2.87 ± 0.35	3.13 ± 0.34	**0.03**
Number of articles discussed sufficient^a^	3.05 ± 0.22	3.00 ± 0.00	3.08 ± 0.28	0.27
Journal club interactive/engaging^b^	4.20 ± 0.71	3.93 ± 0.80	4.38 ± 0.65	0.08
Presenters appropriate/qualified^b^	4.46 ± 0.64	4.47 ± 0.40	4.50 ± 0.66	0.79
Relevance to practice^b^	4.37 ± 0.62	3.93 ± 0.59	4.63 ± 0.59	**< 0.001**
Overall usefulness^b^	4.37 ± 0.50	4.67 ± 0.49	4.50 ± 0.51	0.52
Likelihood to participate in future^b^	4.54 ± 0.71	4.80 ± 0.41	4.38 ± 0.82	0.17
Likelihood to recommend^b^	4.83 ± 0.44	4.67 ± 0.62	4.92 ± 0.28	0.13
Interest in presenting in future^b^	3.64 ± 1.37	4.20 ± 1.01	3.36 ± 1.43	0.14

^a^ A score of 3 represents the most positive response (i.e., “just right”/ideal).

^b^ Scores follow a traditional Likert continuum where 5 indicates the most positive response.

^c^ *p* values < 0.05 are bolded.

## 4 Discussion

This study demonstrates the utility and favorable participant satisfaction of a monthly online journal club for dermatologists and their trainees. Despite the lack of continuing medical education credit and optional attendance, we report impressive attendance rates. The popularity among residents may be due to a perceived lack of cosmetic training and education during dermatologic residency. A survey sent to dermatologic residents in the United States found that only 58% of programs had an encouraging attitude towards cosmetics and 22% had discouraging attitudes [2]. Thus, dermatologic residents may rely more heavily on journal clubs to receive adequate cosmetic education. Despite the engaging and useful content, residents and medical students may not realize the relevance of the topics discussed because they do not have as many opportunities to implement them in their daily practice compared to faculty attendees. The lack of cosmetic procedures and training during residency and discouraging attitudes from programs likely exacerbate this. Medical students may feel the topics are less relevant due to the lack of exposure to dermatology content within their medical school curriculum [3]. Conversely, relevance may be particularly high for faculty due to the large and growing volume of patients seeking cosmetic treatments [4] and the resulting increase in cosmetic procedural volume for dermatologists, trends which residents and medical students may not routinely see or be exposed to.

These differences in faculty and trainee (medical student and resident) responses underscore the importance of multidisciplinary models and learning within academic medicine. The diversity of participant types and breadth of articles selected appeared to contribute positively towards participants' opinions of the journal club and facilitate enriching academic discussions. Prior literature within medical education has shown that journal clubs contribute not only to clinical knowledge but also the acquisition of essential evidence‐based medicine skills, including critical appraisal of evidence and formation of clinical questions [5]. Moreover, journal clubs have been successfully implemented in other medical specialties as well. For instance, journal clubs involving faculty, residents, and medical students within orthopedic surgery [6] and cardiovascular medicine [7] have been associated with improved skills in literature review, clinical thinking, and communication. Taken together, our findings and prior research emphasize the value of multidisciplinary journal clubs for integrating clinical medicine with scientific inquiry.

Limitations of the study include a small number of dermatology residents, who may not be representative of all dermatology residency programs. Additionally, trainees were underrepresented compared to faculty. Categorization of faculty by clinical versus scientific subgroups was not undertaken. Surveys were sent out at the time of the journal club and in the months following the journal club. Respondents who did not fill out the survey at the time of the journal club may have had inaccurate recall of their experience. Lastly, surveys were only distributed to members of the AACD, which may introduce the potential for selection bias and limit the external validity of the findings.

Strengths of the journal club include its accessibility via a web‐based platform, interactive discussion among attendees, impact on clinical practice, and integration of participants across multiple levels of training including medical students, residents, and faculty. Future directions may focus on incorporating resident perspectives when deciding topics or articles to discuss to improve overall relevance.

These findings suggest that a virtual cosmetic dermatology journal club was well‐received and perceived to be a useful method to continue the education of dermatology faculty and trainees.

## Author Contributions

M.A. and B.Y.K.: conceptualization. J.C. and S.A.: methodology, investigation, writing – original draft preparation. J.C.: formal analysis. M.A.: supervision, investigation, writing – reviewing and editing. E.Z., S.S.S., T.G., V.J.L.‐C., D.D., A.A., D.C.C., D.E.M., A.A.H., H.E.‐K., and B.Y.K.: methodology, investigation, writing – reviewing and editing.

## Funding

This research was funded by the Northwestern Department of Dermatology unrestricted research.

## Ethics Statement

The authors have nothing to report.

## Consent

The authors have nothing to report.

## Conflicts of Interest

M.A. and B.Y.K. hold officer positions in the Association of Academic Cosmetic Dermatology. All other authors have no conflicts of interest.

## Data Availability

Data sharing not applicable to this article as no datasets were generated or analyzed during the current study.
